# Incorporation of Alkali-Activated Municipal Solid Waste Incinerator Bottom Ash in Mortar and Concrete: A Critical Review

**DOI:** 10.3390/ma13153428

**Published:** 2020-08-03

**Authors:** Rawaz Kurda, Rui Vasco Silva, Jorge de Brito

**Affiliations:** 1 Department of Civil Engineering, Technical Engineering College, Erbil Polytechnic University, 44001 Erbil, Kurdistan Region, Iraq; rawaz.kurda@epu.edu.iq; 2 Scientific Research and Development Center, Nawroz University, 42001 Duhok, Kurdistan-Region, Iraq; 3 CERIS, Civil Engineering, Architecture and Georesources Department, Instituto Superior Técnico, Universidade de Lisboa, Av. Rovisco Pais, 1049-001 Lisbon, Portugal; rui.v.silva@tecnico.ulisboa.pt

**Keywords:** alkali-activated materials, municipal waste incinerated ashes, strength, durability, construction materials

## Abstract

In the light of one of the most common waste management issues in urban areas, namely the elimination of municipal solid waste (MSW; about 486 kg of the waste per capita were generated in the EU in 2017), this study discusses one technique as an outlet in the construction industry for the by-product of the waste’s incineration in energy recovery facilities (i.e., MSW incinerator bottom ash—MIBA). There have been some investigations on the use of MIBA as partial replacement of cement to be used in cementitious composites, such as concrete and mortars. However, the waste’s incorporation ratio is limited since further products of hydration may not be produced after a given replacement level and can lead to an unsustainable decline in performance. In order to maximize the incorporation of MIBA, some research studies have been conducted on the alkali activation of the waste as precursor. Thus, this study presents an extensive literature review of the most relevant investigations on the matter to understand the material’s applicability in construction. It analyses the performance of the alkali-activated MIBA as paste, mortar, and concrete from different perspectives. This literature review was made using search engines of several databases. In each database, the same search options were repeated using combinations of various representative keywords. Furthermore, several boundaries were made to find the most relevant studies for further inspection. The main findings of this review have shown that the chemical composition and reactivity of MIBA vary considerably, which may compromise performance comparison, standardization and commercialization. There are several factors that affect the performance of the material that need to be considered, e.g., type and content of precursor, alkaline activator, curing temperature and time, liquid to solid ratio, among others. MIBA-based alkali-activated materials (AAM) can be produced with a very wide range of compressive strength (0.3–160 MPa). The main factor affecting the performance of this precursor is the existence of metallic aluminum (Al), which leads to damaging expansive reactions and an increase in porosity due to hydrogen gas generation stemming from the reaction with the alkaline activator. Several approaches have been proposed to eliminate this issue. The most effective solution was found to be the removal of Al by means of eddy current electromagnetic separation.

## 1. Introduction

There have been many efforts to reduce the well-known environmental impacts of the cement industry. The most promising techniques include the use of co-products or by-products as partial replacements of cement. However, their replacement levels are limited as further hydration products are not produced after a given ratio. To overcome this limitation, several studies have risen on alkali-activated materials (AAM), wherein a strong alkaline solution (e.g., NaOH, KOH, and Na_2_SiO_3_) can dissolve amorphous alumina (Al_2_O_3_) and silica (SiO_2_) compounds from a reactive precursor. Alkali-activated mortar and concrete materials have shown a commendable mechanical performance and thus such production techniques have been considered as alternative processes for the production of construction materials despite the yet low acceptance by the industry. Aside fly ash (FA) and ground granulated blast furnace slag (GGBS) have been extensively studied in the production of AAM, and other potential precursors rich in amorphous Al_2_O_3_ and SiO_2_ include:

(i) **Agricultural wastes and aquaculture farming ashes**: rise husk ash [1,2], palm oil fuel ash [3,4,5,6], corn cob ash [7,8], sugarcane bagasse ash [9], straw ash [7,10], forest biomass bottom ash [11], wood ashes [12,13], other agriculture-farming wastes (e.g., alfalfa steam ash, cotton gin ash, com stalk ash and switch grass ash [7,14,15]), and shell wastes [16,17];
(ii) **Industrial waste ashes**: FA [18,19,20,21,22,23,24,25,26,27], coal bottom ash [28], industrials slags [3,20,29,30,31,32,33,34], silica fume [35,36,37,38,39,40,41,42], artificial pozzolans (calcined clays [34,43,44,45], ceramic residues [46,47], sedimentary rocks containing clay minerals and burned bauxites [48,49,50]), natural pozzolans (volcanic tuffs/zeolites [51,52], siliceous such as opal and diatomaceous earth [53,54,55,56,57], and volcanic glasses such as volcanic ashes [58,59,60,61,62], pumice and pumicite [63,64,65];
(iii) **Municipal solid waste ashes**: glass powder [66,67,68,69,70,71,72], sludge ashes [73,74,75,76,77], and municipal solid waste incinerator fly ash [78,79,80,81,82,83,84,85,86], and municipal solid waste incinerator bottom ash [86,87,88,89,90,91,92,93,94,95,96,97,98,99,100,101,102,103,104,105,106,107,108,109].

Regarding municipal solid waste (MSW), in 2012, the total global production of waste amounted to about 1.3 billion tons and, in 2025, this value is expected to reach up to 2.2 billion tons, representing 1.42 kg/person/day [110]. One of the most effective measures to treat MSW is by means of incineration with energy recovery, which is capable of reducing the initial weight sent to landfill by up to 75% [111,112]. However, this incineration process is responsible for two by-products: MSW incinerated fly ashes and bottom ashes (MIBA). The latter waste is then processed to be used in construction, but, because of low demand, most of it is generally left unused in sanitary landfills. Accordingly, one approach that has been considered as an outlet for MIBA is by mixing it with a strong alkaline solution to produce a construction-fit AAM. Therefore, this paper presents a literature review on the topic, wherein an extensive database was built to synthesize, identify, and evaluate the existing research on the mentioned topic. After providing an explanation of the methodology on how the review was conducted, it presents a general perspective of the physical and chemical characteristics of untreated MIBA, how these may vary and their importance when performing alkali activation. Subsequently, the paper presents the main findings in the literature regarding alkali-activated MIBA as sole/partial precursor. Different topics of interest were identified in the literature including the behavior of alkali-activated MIBA in the fresh and hardened states, the optimization of the alkali activator and influence of the thermal curing regime from a performance maximization perspective and also improving treatments to MIBA. This literature review also provides a brief depiction of the role of alkali-activated MIBA in the solidification/stabilization of hazardous compounds.

## 2. Methodology

The following literature review, which concerns MIBA-based AAM, focuses on the parameters affecting the mechanical and durability-related performance of pastes, mortars, and concrete. The literature search was made using mostly the Web of Science and Scopus search engines. For each database, the same search options were repeated using combinations of the following keywords:

- Bottom ash;
- Concrete;
- Geopolymer;
- Hybrid cements;
- Hydrothermal synthesis;
- Incineration;
- Incinerator bottom ash;
- Inertization;
- Mortar;
- Municipal solid waste;
- Paste;
- Stabilization/solidification.

Several boundaries were established to find the related studies for further inspection. The validity of the selected papers was specified by analyzing the title and abstract. Thereafter, the non–relevant studies were removed after evaluating the relevance of the materials and methodology of the research studies and how they were related with the study in question. For that purpose, two main criteria were chosen in order to demonstrate whether a publication was relevant to this research work. The chosen studies met the following criteria:

- Papers explicitly concerning alkali-activated paste, mortar, and/or concrete made with MIBA as the sole precursor or MIBA blended with other supplementary cementitious materials (SCM) or ordinary Portland cement (OPC);
- MIBA must be used as a precursor (binder). Other applications of MIBA e.g., studies on AAM containing MIBA as aggregate were excluded.

## 3. Physical and Chemical Characteristics of MIBA

**Figure 1:**
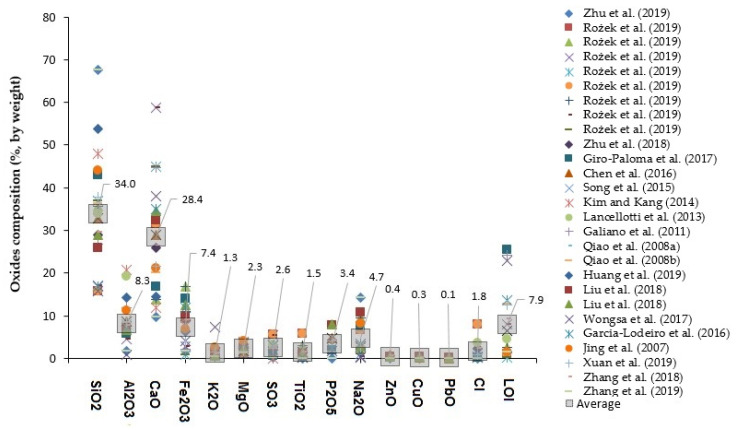
Oxides composition of MSW incinerator bottom ash (MIBA) from various studies [88,89,90,91,92,93,94,95,96,97,98,99,100,101,102,103,104,105,106,107,108,109].

It is widely known that understanding the oxide composition via X-ray fluorescence (i.e., SiO_2_, Al_2_O_3_, CaO, Fe_2_O_3_, K_2_O, MgO, SO_3_, TiO_2_, P_2_O_5_, Na_2_O, ZnO, CuO, PbO, and Cl, and loss on ignition) plays a vital role in identifying the suitability of any precursor for the production of AAM. This test, in parallel with quantitative X-ray diffraction analysis, allows identifying the potential of any precursor based on the quantity of amorphous aluminosilicate phases. The oxide composition is indicative of the materials’ contents, but not of its pozzolanicity/reactivity since many of its phases are crystalline. Alkali activators, such as sodium hydroxide and sodium metasilicate, react with amorphous Al_2_O_3_ and SiO_2_ present in the solid precursor, resulting in a solid inorganic polymer showing properties comparable to those of hydrated cement. It is widely known that the chemical composition of the precursor has a considerable influence on the performance of its resulting AAM. Figure 1 presents the oxide composition of MIBA from several recent studies [67,88,89,90,91,92,93,94,95,96,97,98,99,100,101,102,103,104,105,106,107,108] to complement the study carried out by some of the authors in the past [112]. The results show that SiO_2_ usually is the predominant component, followed by CaO, Al_2_O_3_, Fe_2_O_3_, Na_2_O, P_2_O_5_, SO_3_, MgO, and CI with average contents of 34%, 28%, 8%, 7%, 5%, 3%, 3%, and 2% respectively, and the other components are less than 1.45%. However, there was a notable scatter, which can be explained by the considerable variability of MSW.

Generally, a high amount of SiO_2_ can be expected from MIBA, though not necessarily in an amorphous nature. Si-bearing compounds usually come from glass cullet (e.g., end-of-life consumer glass containers), sand, cementitious materials, ceramic products, among several others. It has been suggested that MIBA can be defined as a silica-based powder because of its high SiO_2_ content coming from discarded soda-lime-silicate glass [91]. A high amount of CaO is typically observed in MIBA and this compound most likely comes from improperly discarded construction and demolition waste comprising high amount of cementitious materials. Most of the aluminum-containing phases in MIBA are likely to be in its purest metallic form, which is known to have corrosion reactions in alkali environments. High amounts of Fe_2_O_3_ probably from discarded ferromagnetic metals are also characteristics of MIBA. Although separation stages for ferrous and non-ferrous metals (eddy current) are applied during the treatment of MIBA, these are not entirely effective resulting in some contamination of the product. The most common magnetic separation techniques are cross-belt magnetic, drum magnetic, and magnetic pulley [113]. Further details regarding the magnetic separation can be seen in the study of Joseph et al. [114]. Table 1 provides a comparison with the oxide composition for Portland cement, with minimum, maximum, and mean values of the main chemical composition. It promptly highlights the greater content and importance of CaO- and SiO_2_-containing phases in a cementitious binder as they lead to the formation of C-S-H (major contributor to strength). The presence of Al_2_O_3_ is typically associated with the formation of AFm phases [115], which increases durability performance [116,117]. The presence of strongly alkaline metals (i.e., sodium and potassium—Na_2_O and K_2_O) are limited in content in conventional (must not be higher than 1% for OPC concrete [118]) as they react with active SiO_2_ of the aggregates and cause disruptive alkali silica reactions. The resulting gel can expand and cause extensive cracking and internal failure at longer ages in conventional concrete. However, such limit is not imposed for AAM, as they participate in polymerization reactions.

**Figure 2:**
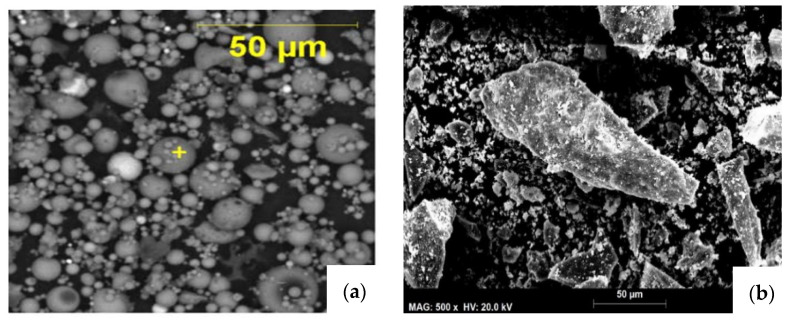
SEM images of (**a**) FA and (**b**) MIBA particles.

After incineration, MIBA presents particle size distribution like that of “all-in” aggregates. However, previous studies have shown that, after being submitted to a milling process, MIBA can present a particle size distribution similar to that of Portland cement [94,120,121,122]. According to the database made by some of the authors [112], the ranges of specific gravity and moisture content of MIBA were found to be 2.24–2.78% and 12–18%, respectively. Scanning electron microscopy (SEM) has shown that, unlike coal FA particles (Figure 2a—smooth glassy spherical particles [121]), MIBA particles are irregular and rough because of the relatively low incineration temperatures (~800 °C) that are under the melting point of the majority of minerals [99]. These irregular and rough surfaces have been identified as a result of the formation of gasses during the incineration process, resulting in a porous microstructure Figure 2b.

It may not be reliable to compare the threshold values defined for the chemical composition of cement and those of any type of precursor used to produce AAM. However, the threshold values of cement can be used as a benchmark and provide a preliminary idea about the performance of the precursor. According to EN 197-1 [123], which presents the standard composition, specifications and conformity criteria for common cements in the European Union, the percentage of SO_3_ in cement must be up to 4.5% as higher contents lead to internal disruption. However, this limit may not make sense in AAM since these are much more resilient to sulphate attack. The range of MgO in cement should be around 1–4% in order to control the expansion during the hydration process, but, in AAM, the mechanism of MgO hydration and consequent expansion is not entirely known and thus the limit for this constituent may be further increased. Furthermore, TiO_2_ is a photo-catalytic component and may not directly affect the chemical reaction of the paste [124]. Moreover, P_2_O_5_ in cement decreases the early strength of the paste when its content is higher than 2.25% of clinker mass because it reacts with 2CaO and SiO_2_ and decreases C-S-H as a result. Normally, CuO and ZnO need to be determined to understand the risk of leaching potential of paste due to Cu and Zn metals. Additionally, ZnO, CuO, and PbO can be considered as retarders (delaying the hydration). Their mass concentration should be less than 0.3% [125], 1% [126], and 0.04% [127,128,129], respectively, for conventional concrete, but additional information is required for AAM.

## 4. Alkali-Activated Materials with MIBA as Sole/Partial Precursor

An alkali activator is used to react with the precursor (solid aluminosilicate) under alkaline conditions to produce hardened AAM, which is made in a complex “alkali-alkali earth-aluminosilicate and/or hydrous alkali-aluminosilicate” phase [130]. MOH and M_2_SiO_3_ (M is either K or Na) are the most commonly used activators. Other activators include Na_2_CO_3_ and Na_2_SO_4_ [131]. CaO, Ca(OH)_2_, and MgO are also identified as potential activators, though less used [131].

There are two main procedures to produce AAM, either by adopting a one-part approach (dry alkali activator mixed with water and precursor at the same time) or the conventional (two-part) approach (activator prepared previously and then mixed with the precursor). Both procedures were considered in this study.

Table 2 presents a summary of the main studies on the alkali activation of MIBA as sole/partial precursor of pastes, mortars, or concrete. Although many of them studied MIBA as the only precursor, there were some incorporating several types of SCM (e.g., FA, GGBS) to improve the performance of AAM, since MIBA typically presents lower content of amorphous phases. It was perceived that, in most studies, AAM samples were subjected to thermal curing (normally at 60–80 °C) considering the materials’ inherent endothermic reaction for strength to develop. Concerning the alkali activators, most studies have used only NaOH, or Na_2_SiO_3_ mixed with NaOH to prepare the alkaline solutions. From a general perspective, the literature on this subject is still scarce and just starting as most studies were carried out on mortars and pastes. Existing studies are normally on the mechanical performance of activated specimens with a focus on optimization of the alkaline activator and analysis of the microstructure. Some attention has also been paid to the leachability and toxicity of AAM containing MIBA.

### 4.1. Fresh, Mechanical and Durability Performance

**Figure 3:**
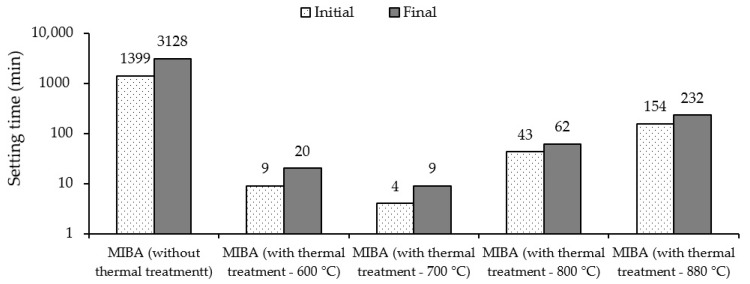
Effect of thermally treated and untreated MIBA on the setting time of pastes (adapted from Qiao et al. [107]).

Surprisingly, the early age performance (e.g., slump and setting time) of MIBA-based AAM has not been extensively studied in view of the shape of particles and their lower reactivity. Figure 3 presents the setting time of mixes of MIBA previously subjected to a high temperature treatment (600–880 °C) and activated with Ca(OH)_2_ [98]. This treatment led to the production of a higher reactivity-exhibiting material presenting a fast setting when compared to the untreated MIBA. It was associated with the formation of aluminate phases (carbonate-sulphate AFm-type phase). In comparison with OPC pastes, the setting times of alkali-activated pastes made with untreated MIBA are likely to be much higher [98,107]. In the study of Garcia-Lodeiro, Carcelen-Taboada, Fernández-Jiménez and Palomo [103], a hybrid cement was manufactured by blending 40% of alkali-activated MSW ashes and 60% of OPC. About 5% of CaSO_4_ was successfully used to control the system’s setting.

Zhu et al. [90,93] studied the characteristics of AAM using the glass fraction collected from MIBA. Aqueous Na_2_SiO_3_ (H_2_O—65%, SiO_2_—27%, and Na_2_O—8%) and NaOH (14 M) were used as activators in mixes with a l/s ratio of 0.5 and subsequently cured at 75 °C and 98% RH for 3 days. The 3-day compressive strength of the samples with MIBA was around 3 MPa, whereas specimens made with the glass fraction alone presented compressive strengths close to 70 MPa. This increase was due to the use of milled glass only, which increased the amount amorphous material capable of strongly reacting with the alkaline solution [132,133]. Nuclear magnetic resonance spectral analysis showed that the structural evolution, from the precursor to the gel, presented higher nano-structural connectivity than that of the precursor and that both the amount and polymerization degree of the gel increased with increasing SiO_2_/Na_2_O ratio from 0 to 1.2 [88]. Increasing the SiO_2_/Na_2_O ratio of the alkaline solution from 0 to 2.5 led to a near 60 MPa increase in compressive strength. By means of the salicylic acid and methanol method, the authors determined that about 20% by mass of the activated binder is composed of C-S-H and Na_2_Ca(CO_3_)_2_·2H_2_O (hydrated sodium calcium carbonate or pirssonite) [134]. Thermogravimetric analysis suggested a 5% CaCO_3_ content. Nuclear magnetic resonance suggested 17% of aluminosilicate gels by mass coexisting with C-S-H. The former showed a similar nanostructural connectivity to that of aged alkali-activated FA with high Si/Al ratio, because of the low content of Al in MIBA.

Huang et al. [135] studied the behavior of alkali-activated concrete containing 56% MIBA and 44% GGBS as precursors and varying amounts of NaOH and sodium silicate solutions. 28-day compressive strength reached almost 50 MPa. After having subjected the specimens to an accelerated carbonation for 60 days in a chamber with 20% CO_2_ with a relative humidity of 70%, the authors determined that specimens with a pondered amount of silicate solution and NaOH as activators yielded a lower carbonation depth in comparison with that of specimens using NaOH as the sole activator.

**Table 2:** Studies related to alkali-activated construction materials made with MIBA.

Study	Sample	Tests										
		Mechanical Performance	Toxicity	XRD ^a^	SEM ^b^	FT-IR ^c^	XRF ^d^	Density-Porosity	Conductivity—Heat	Setting Time	pH	Others
Zhu et al. [134]	Paste	X	-	X	-	X	-	-	-	-	-	NMR ^e^
Cristelo et al. [136]	Paste	X	X	X	X		X	-	-	-	-	-
Rożek et al. [89]	Paste	X	X	X	X	X	X	X	-	-	-	Raman spectra
Zhu et al. [90]	Paste	X	-	X	-	X	X	X	-	-	-	-
Giro-Paloma et al. [91]	Paste	-	X	X	X	X	X	-	-	-	X	TGA ^f^
Chen et al. [92]	Paste	X	X	X	X	X	-	X	-	-	-	-
Zhu et al. [88]	Paste	X	-	-	-	X	X	X	-	-	-	NMR ^e^
Song et al. [94]	Paste	X	-	X	X	-	-	X	-	-	-	Gas production; shrinkage
Kim and Kang [95]	Paste	X	-	X	X	-	-	-	-	-	-	-
Lancellotti et al. [96]	Paste	-	X	X	X	X	-	-	X	-	-	EDS ^g^
Krausova et al. [109]	Paste	-	X	X	X	-	-	X	-	-	X	-
Galiano et al. [108]	Paste	X	X	-	-	-	-	-	-	-	X	-
Onori et al. [97]	Paste	X	X	-	X	X	-	X	-	-	X	TGA ^f^
Qiao et al. [98]	Paste	X	-	X	X	-	X	-	-	X	-	Gas production
Qiao et al. [107]	Paste	X	X	X	X	-	-	X	-	X	-	-
Huang et al. [99]	Mortar	X	-	X	X	X	-	-	-	-	-	Active silica content; EDS g
Huang et al. [100]	Mortar	X	X	X	X	X	-	-	-	-	X	-
Huang et al. [137]	Mortar	X	-	X	-	X	-	-	-	-	-	TGA ^f^
Liu et al. [101]	Mortar	X	-	X	-	-	-	X	-	-	-	Release of gas
Wongsa et al. [102]	Mortar	X	-	X	X	X	-	X	-	-	-	-
Garcia-Lodeiro et al. [103]	Mortar	X	X	X	X	-	-	-	-	-	-	-
Jing et al. [104]	Mortar	-	X	X	X	-	-	X	-	-	-	-
Penilla et al. [105]	Mortar	-	-	X	X	X	-	-	-	-	-	-
Huang et al. [135]	Concrete	X	-	X	-	X	-	-	-	-	X	Carbonation
Xuan et al. [106]	Concrete	X	-	X	X	X	X	X	X	-	-	EDS ^g^

^a^ XRD—X-ray diffraction; ^b^ SEM—scanning electron microscope; ^c^ FT-IR—Fourier transform infrared spectroscopy; ^d^ XRF—X-ray fluorescence; ^e^ NMR – nuclear magnetic resonance; ^f^ TGA—thermogravimetric analysis; ^g^ EDS—energy dispersive X-ray spectroscopy.

**Figure 4:**
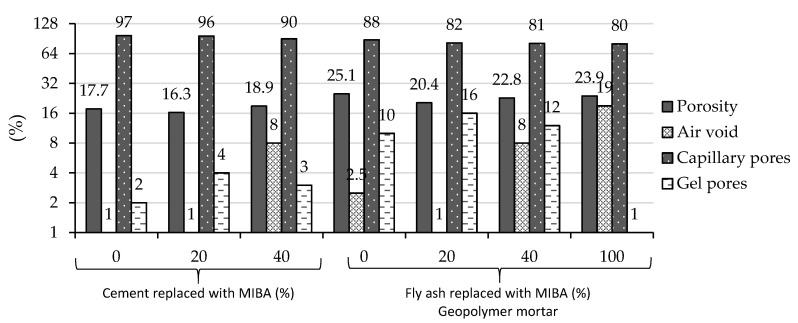
Effect of MIBA on the microstructure of cement and geopolymer mortar (adapted from Wongsa et al. [102]).

Wongsa et al. [102] produced alkali-activated mortars using MIBA and type C FA as precursors (proportions by weight of total precursor of 0/100, 20/80, 40/60, and 100/0). MIBA was ground by ball mill to a maximum particle size of 45 µm. 10 M NaOH and aqueous Na_2_SiO_3_ (12.53% Na_2_O, 30.24% SiO_2_, and 57.23% H_2_O) solutions were used as activators. Optimum 28-day compressive strength was observed for specimens with 20% MIBA and 80% FA (about 53 MPa), whereas 100% MIBA led to a value of 10.6 MPa. Apart from the clear trend in the pore size distribution results, porosity, compressive strength, and SEM, the incorporation at 20% of MIBA with 80% FA was deemed as optimum for use as a precursor in alkali-activated mortars. The authors also compared the porosity, air voids, capillary pores, and gel pores of the cement-based and alkali-activated specimens (Figure 4). As mentioned, the mix proportion of 20% MIBA with 80% FA was found to be optimum as it increased homogeneity and density, leading to lower amount of air voids and high gel pore content.

**Figure 5:**
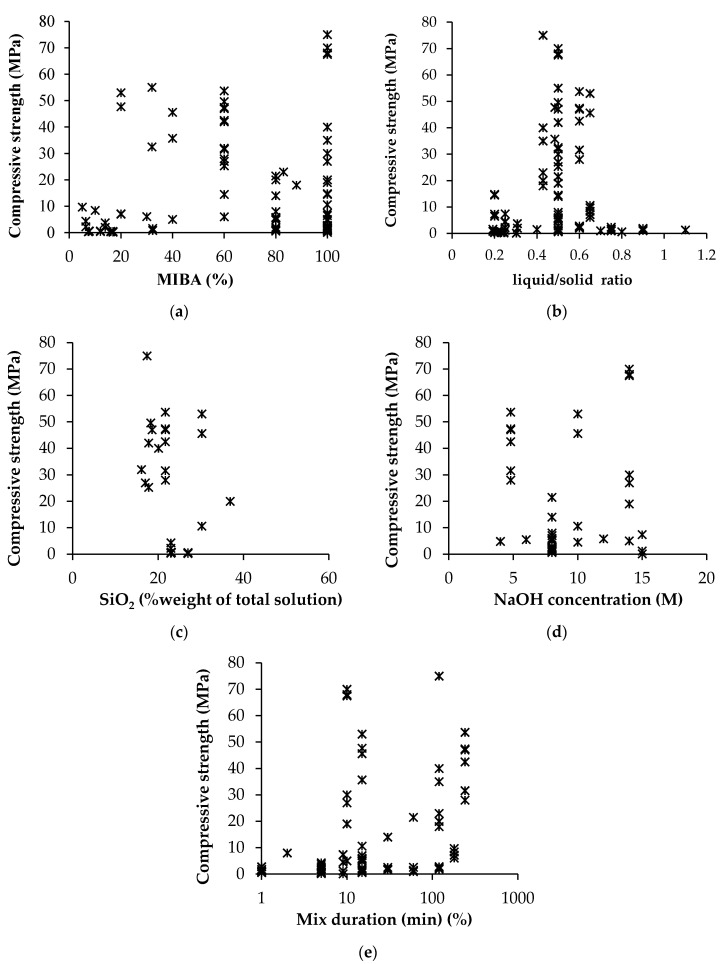
Compressive strength vs. (**a**) MIBA incorporation ratio, (**b**) liquid/solid ratio, (**c**) total SiO_2_ quantity in solution, (**d**) NaOH concentration and (**e**) mix duration.

Figure 5 presents a simple organization of several factors influencing the compressive strength of mortars, sourced from the studies mentioned in Table 1, to understand existing trends. It was perceived that MIBA-based AAM can be produced with extremely varying compressive strength (0.3–75 MPa) regardless of the incorporation level. Aside the inherent variability of the characteristics of MIBA as well as its beneficiation treatments and milling processes, the strength of MIBA-based AAM also depends on several factors related to the mix design, including: liquid to solid ratio; (K, Na)OH content; SiO_2_ to Na_2_O ratio, mixing time. Lower liquid to solid ratio is expected to result in improved mechanical performance, as it will increase the proximity of reaction products thereby facilitating their physical and chemical binding.

Generally, a higher content of the alkaline activator (in this case, NaOH as it is the most widely studied activator) leads to a greater and faster dissolution of amorphous aluminosilicate particles, with subsequent nucleation and growth. Although Figure 5d does not infer it, individual studies in the literature have shown that there is a maximum strength development for an optimum content of NaOH, after which it a decline in performance is observed.

In the particular case of MIBA, the duration of the mixing process is also a key factor influencing the performance of AAM, as the precursor typically has metallic Al, which reacts in contact with the OH-rich solution, releasing H_2_ gas. This causes the formation of air pockets in the material subsequently leading to a considerable decline in performance. From a practical perspective, the performance of MIBA-based AAM improves with increasing mixing duration.

**Figure 6:**
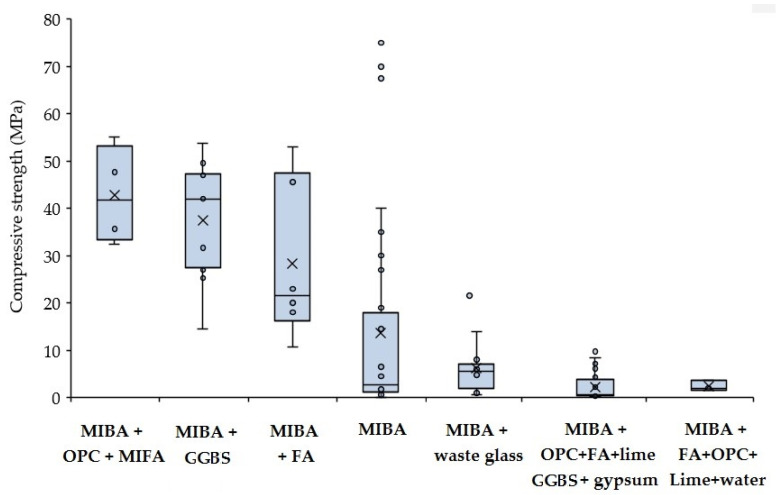
Effect of various types of precursor on alkali-activated materials (AAM).

As mentioned, apart from the alkali activator, the type of the precursor is one of the main factors affecting the strength of AAM. Figure 6 shows a box plot chart of the average values of the compressive strength of alkali-activated MIBA mixed with different types of SCM. Generally, the content of MIBA in AAM varies and is typically complemented with FA or GGBS. As expected, it infers that the mechanical performance can significantly improve by incorporating GGBS. This SCM is widely regarded as a high-performance binder in the production of AAM [138]. FA is also capable of imparting high strength for given incorporation levels. MIBA alone, when untreated, generally produces mixes with relatively low performance.

**Figure 7:**
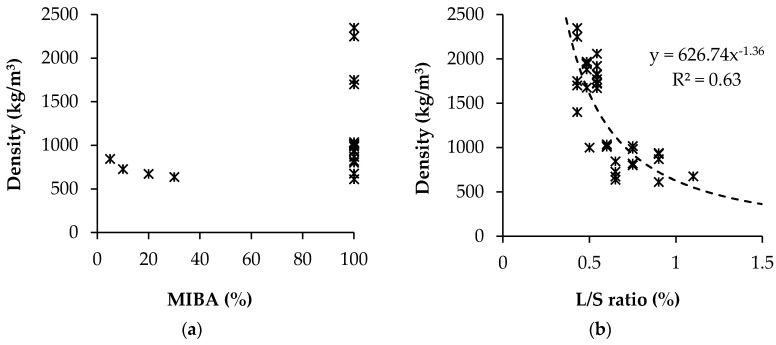
Density of AAM vs. (**a**) MIBA incorporation ratio and (**b**) liquid to solid ratio.

Literature regarding the density of MIBA-based AAM is scarce. Generally, the density of alkali-activated MIBA is relatively low (612–1036 kg/m^3^) because of the air voids generated during the reaction of the precursor (i.e., corrosion of metallic Al) and the alkaline solution (Figure 7a). As expected, the density of AAM mixes decreases with increasing liquid to solid ratio (Figure 7b).

**Figure 8:**
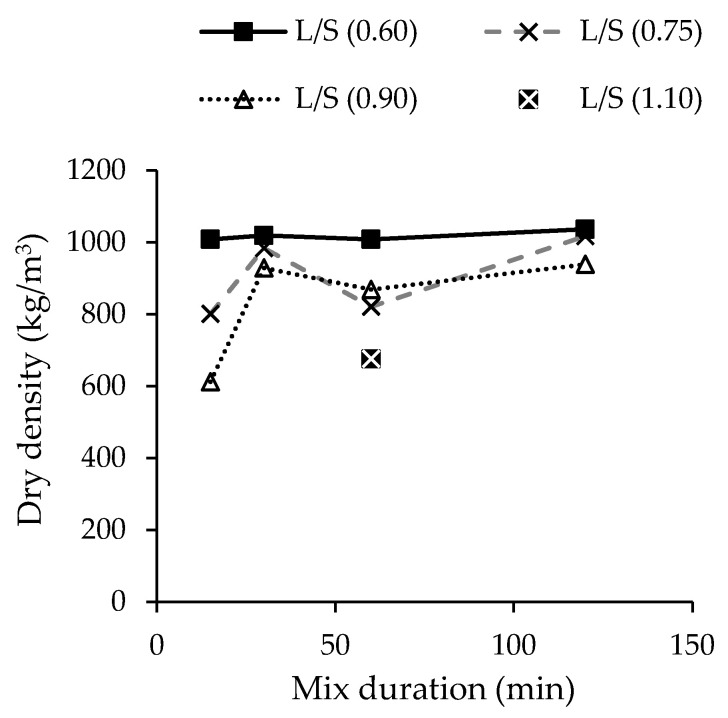
Influence of liquid to solid ratio and mix duration on density of alkali-activated MIBA (adapted from Chen et al. [92]).

Chen et al. [92] studied the dry density of alkali-activated paste made with MIBA. The authors reported that the lower density (600–1000 kg/m^3^) of the sample is related to the H_2_ gas from the reaction between the precursor’s Al fraction and the alkaline solution. In addition, the authors studied the effect of liquid to solid ratio and mix duration on the dry density and porosity of the samples. The results show that the density increased with increasing mixing time (Figure 8). Further details regarding the issue of Al can be seen in the study of Song et al. [94], and Zhe et al. [88,93] also observed that the dry density of alkali-activated pastes containing MIBA was low (900–1100 kg/m^3^) because of H_2_ gas formation.

**Figure 9:**
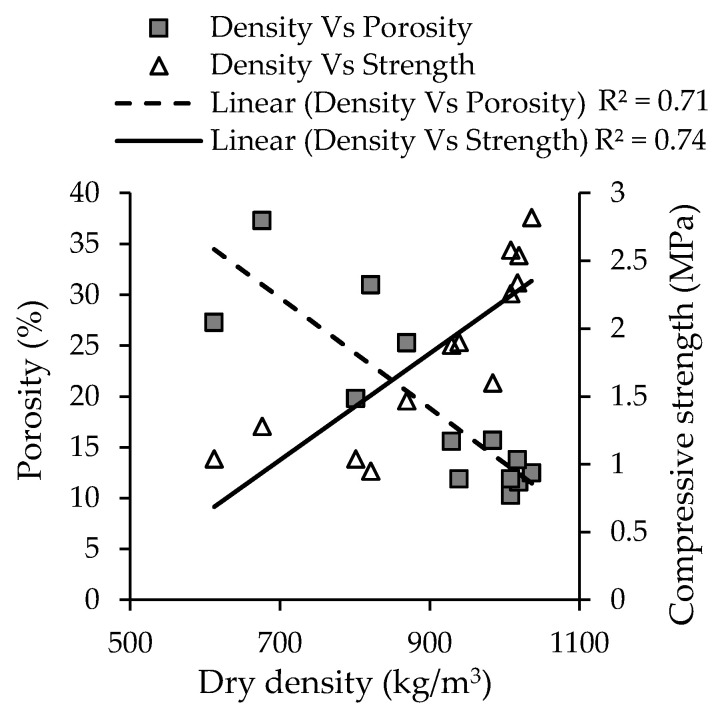
Relationship between microstructure and strength of the alkali-activated MIBA (adapted from Chen et al. [92]).

Figure 8 also shows that the dry density of the samples increased with decreasing liquid to solid ratio. The authors reported that higher liquid to solid ratio not only increased porosity but also accelerated the rate of production of H_2_ gas. Figure 9 shows the relationship between the dry density, porosity, and compressive strength of the pastes. The results show that there is a strong relation between strength and both porosity and density of the samples. Therefore, it is important to solve the issue of hydrogen formation in order to obtain high strength materials.

Qiao et al. [107] obtained alkali-activated MIBA with a bulk density of 2000 kg/m^3^ by pressing the sample. Rożek et al. [89] also showed the advantage of pressing technique in terms of a denser microstructure (2350 kg/m^3^). Jing et al. [104] showed a relationship between bulk density, tensile strength with the compaction pressure on the alkali-activated mortars with MIBA and confirmed the necessity for a pressing technique.

**Figure 10:**
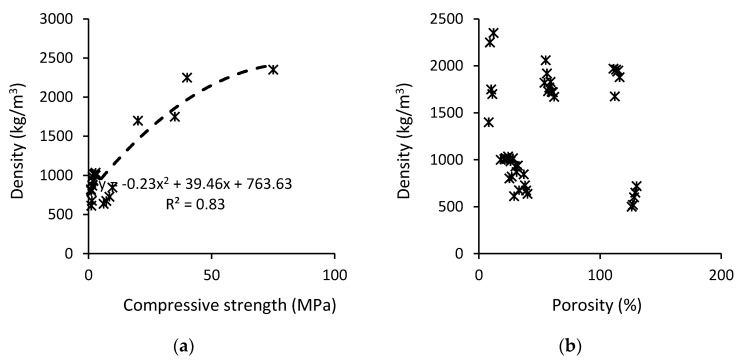
Relationship between density and (**a**) compressive strength and (**b**) porosity of AAM.

A collation of the results of on density vs. compressive strength and porosity can be seen in Figure 10. As expected, it shows that there is a strong relationship between the compressive strength and density of MIBA (R^2^ ≈ 0.83). Contrary to expectations, no strong correlation between density and porosity was found (Figure 10b).

### 4.2. Effect of Alkali Activator Composition

The composition of the alkaline activator is one of the most important factors influencing the performance of MIBA-based AAM. It has been reported that the Si/Al and Na/Al ratios must be in the ranges of 1.8–2.5 and 0.9–1.2, respectively [139,140] to achieve optimum performance. This is done by adjusting the quantities of NaOH and Na_2_SiO_3_ (most typically used activators) to match the specific chemical composition of the precursor. It has been stated that the normal range of alkali component is 5–10% of total mass of the precursor and with a molar concentration of NaOH between 2 M and 14 M [130,141]. Generally, the influence of the alkali activator on AAM depends on its quantity and concentration as well as the precursor’s reactivity. Table 3 presents a collation of the range and optimum values of the Na_2_SiO_3_/NaOH ratio, NaOH concentration, and silica modulus (SiO_2_/Na_2_O) in AAM using different precursors to provide a benchmark for MIBA-based mixes. A higher Na_2_SiO_3_/NaOH ratio is known to decrease the workability of mortars because of the higher viscosity of the solution. Higher concentrations of NaOH may increase the setting time of AAM [141]. The SiO_2_/Na_2_O ratio allows an understanding of the degree of polymerization of silicate gel phases, which generally increases with for higher ratios [88]. According to Table 3 and also to the findings in the literature [130], the optimum SiO_2_/Na_2_O ratio is typically between 1.0 and 1.5. Concerning the optimum NaOH concentration, it varies considerably depending on the precursor. There is a consensus in the literature that 10–15 M NaOH typically results in enhanced mechanical performance as the solution exhibits a higher pH level leading to a more effective dissolution of aluminosilicate particles. Greater concentrations can lead to the precipitation of less resistant phases thereby diminishing overall strength.

Maldonado-Alameda et al. [142] studied the amount of SiO_2_ and Al_2_O_3_ extracted from MIBA with different particle sizes (2 mm to 30 mm) using NaOH solutions with concentrations of 2 M, 4 M, and 8 M. The amount of amorphous content varied between 44% and 70% depending on the size fraction; 4–8 mm contained the highest content probably because it corresponded to typical size of glass cullet (corroborated with XRD and XRF analyses). Regardless of the size fraction, there was a greater percentage of extracted SiO_2_ and Al_2_O_3_ for mixes using an 8 M NaOH solution.

Regarding the mechanical performance of mixes in the hardened state, even though there were some attempts to optimize the mix design in view of enhanced performance of alkali-activated MIBA, there are few definite findings. Chen et al. [92] produced aerated AAM with MIBA as a precursor. Most of the NaOH-based activator’s concentration was of 8 M, but concentrations of 2 M, 4 M, and 12 M were also evaluated. Liquid/solid (l/s) ratios varied between 0.6 and 1.1, and mix duration ranged from 15 to 120 min to potentiate metallic Al corrosion and thus H_2_ gas production. Concentration of 8 M and mixing for 60 min were found to be optimum in terms of compressive strength (maximum compressive strength of 2.82 MPa) and porosity. Further increasing mixing time resulted in enhanced performance due to H_2_ release rather than leaving it entrapped in the fresh mix.

**Table 3:** Influence of chemical activator on the performance of alkali-activated concrete.

Studies	Precursor *	Na_2_SiO_3_/NaOH Ratio		NaOH Concentration (M)		Silica Modulus (SiO_2_/Na_2_O ratio)		Compressive Strength (MPa)
		Range	Optimum	Range	Optimum	Range	Optimum	
Görhan and Kürklü [143]	FA	-	-	3–9	6	3.0	-	12–23
Sukmak et al. [144]	FA	0.4–2.3	0.7	10	-	-	-	4–14
Somna et al. [145]	FA	-	-	4.5–16.5	14.0-	-	-	7–23
Ridtirud et al. [146]	FA	0.33–3.0	1.5	7.5–12.5	7.5	-	-	25–45
Guo et al. [147]	FA	-	-	-	-	1.0–2.0	1.5	5–63
Law et al. [148]	FA	-	-	10	-	0.75–1.25	1.0	39–57
He et al. [149]	RHA	-	-	2–6	2	-	-	8–15
Nazari et al. [150]	RHA	2.5	-	4–12	12	-	-	20–30
Songpiriyakij et al. [151]	RHA	0.5–2.5	-	14, 18	18	0.13–0.27	0.13	22–56
Detphan and Chindaprasirt [1]	RHA	1.9–5.5	4.0	-	-	-	-	15–40
Salih et al. [6]	POFA	0.5–3.0	2.5	10	-	-	-	7–32
Yusuf et al. [152]	POFA	-	-	10	-	0.92–1.64	0.92	65–69
Ahmari and Zhang [153]	MS	-	-	10–15	15	-	-	4–34
Wongsa et al. [102]	MIBA	1	-	10	-	-	-	10.6
Zhu et al. [90]	MIBA	0.5	-	8	-	-	-	2.8

* FA—fly ash; RHA—rice husk ash; POFA—palm oil fuel ash-based; MS—hematite mine tailings.

Huang et al. [99] studied the performance of mortars containing 60% MIBA and 40% GGBS focused on the presence of reactive SiO_2_ both from the precursors and from the alkaline activator NaOH and Na_2_SiO_3_. The compressive strength of the alkali-activated samples without Na_2_SiO_3_ was relatively low (~15 MPa after 28 days) because of low active SiO_2_ content, which affected the nucleation and growth of C-A-S-H and C-S-H. The mechanical performance improved (~50 MPa after 28 days) after incorporating about 26% of Na_2_SiO_3_ solution (SiO_2_/Na_2_O ratio of 1.04). Higher quantity of Na_2_SiO_3_ solution hindered the strength development due to excess of Na and Si in the pore solution, causing the precipitation of magadiite (NaSi_7_O_13_(OH)_3_·4(H_2_O)), which is known for its instability and low hardness.

Cristelo et al. [136,154] produced pastes using varying concentrations of NaOH (4–12 molal) in the alkaline activator as well as with the incorporation of sodium silicate (SiO_2_/Na_2_O weight ratio of 2.0) for the activation of MIBA and/or fly ash from MSW incineration. Liquid to solid ratios varied between 0.35 and 0.50. Compressive strength of mixes with NaOH alone were low (between 1.5 and 3 MPa) but increased with the use of sodium silicate (over 10 MPa). As before, the decline in performance was mostly due to the extensive expansion caused by the reaction of Al with the NaOH solution. When exposed to wetting and drying cycles, specimens with a 4 m NaOH activator exhibited quite low stability, demonstrating severe mass loss after each cycle.

Huang et al. [155] studied the effect of increasing NaOH content of mortars containing alkali-activated MIBA and GGBS. The sodium silicate solution (9.65% Na_2_O, 25.22% SiO_2_, and 65.13% H_2_O) content was constant and about 27% of the binder’s weight, to which water and NaOH were added. Increasing amount of NaOH content led to higher pH levels, which translated to a more effective dissolution of aluminosilicate phases and thus improved strength development (47.4 MPa for mixes with NaOH content of 5.55% that of the weight of the precursors). 28-day compressive strength of 27.9 MPa and 36.5 MPa was observed for mixes containing about 2% and 11% of NaOH, respectively, indicating optimum levels in between. By means of a conversion rule between free and “union” alkali, the authors demonstrated that, for a given amount of total alkali incorporated in the mortars, there was a considerable loss of alkali in mixes made with sodium silicate alone to the external environment (diffusion mechanism to achieve equilibrium with an efflorescence phenomenon). The amount of “union” alkali, which corresponds to that of resulting of the polymerization process, was higher for mixes with a 5.55% NaOH content.

### 4.3. Influence of Thermal Curing Regime on the Performance of the MIBA-Based AAM

The thermal curing of AAM is considered as one of the most important strength-developing parameters because of the inherent endothermic nature of the reaction. Normally, AAM samples are subjected to a relatively high curing temperature for a short period of time (generally, 60–80 °C for 10–72 h), followed by curing at room temperature until testing age. Given the importance of this parameter from a practical point of view, there have been some studies on the effect of varying the curing temperature and relative humidity (RH) on the mechanical performance of alkali-activated MIBA. Galiano et al. [108] produced AAM with FA and MIBA as precursors (74% and 26%, by weight, respectively, of the total binder) and various contents of NaOH, KOH, Na_2_SiO_3_, and K_2_SiO_3_ as activators. Specimens cured at 60 °C exhibited higher strength than mixes cured at room temperature. Almost all mixes showed 28-day compressive strength below 1 MPa. Though undisclosed by the authors, this low performance was probably due to the formation of H_2_ gas from Al reaction with the OH^−^-rich solution.

Huang et al. [100] assessed the influence of different curing methods on the performance of mortars containing 60% MIBA and 40% GGBS. The methods consisted of natural curing (5–20 °C and 60% RH), standard curing room (20 ± 2 °C and >95% RH), seal curing (20 ± 2 °C and >95% RH), steam curing (80 ± 2 °C and >100% RH), and soaking curing (20 ± 2 °C and 100% RH). 28-day compressive strength varied between 28 MPa and 53 MPa, depending on the curing method. The authors observed that natural and soaking curing methods yielded lower strength because of leaching of OH-, which led to a reduction of pH and thus lower dissolution of active components. Optimum performance was observed in samples cured in high RH environment, yet with low contact with external water (i.e., seal and standard curing methods).

### 4.4. Performance-Enhacing Treatments

#### 4.4.1. Thermal Treatment

In spite of MIBA coming from MSW incineration power plants, wherein the waste is treated at high temperatures, the combustion process is largely ineffective and yields a bottom ash with considerable loss on ignition (LOI) due to high content of unburnt organic carbon. For this reason, there have been studies focusing on subsequent thermal treatment of MIBA. In the study of Qiao et al. [107], treatment temperatures varied between 600 °C and 880 °C. For this treated MIBA, Ca(OH)_2_ was used as the alkaline activator. Untreated MIBA led to a 28-day compressive strength of 0.6 MPa, whereas AAM with MIBA treated at 700 °C exhibited a compressive strength of almost 3.0 MPa. Despite some of the mineralogical changes after the heat treatment, including gehlenite (Ca_2_Al_2_SiO_7_), wollastonite (CaSiO_3_), and mayenite (Ca_12_Al_14_O_33_), which may have led to some strength increase, the main cause of the low strength was due to the formation of H_2_ gas from the reaction of metallic Al leading to a macro-porous structure.

Krausova et al. [109] mixed various ratios of glass powder (10–30%) with fly ash and bottom ash from MSW incineration (equal mass proportions) and then thermally treated at 700 °C and 800 °C. The alkaline activator was composed of Na_2_SiO_3_ (N_2_O = 9.5%; SiO_2_ = 29%; H_2_O = 61.5%) and NaOH solution with a concentration of 45%. The results showed that the incorporation of 10% glass powder, in activated ashes previously treated with a regime of 700 °C for 1 h, yielded maximum dry density values. Lowest porosity and water absorption were achieved by activated ashes containing 30% glass powder and were treated at 800 °C for 1 h. These findings alongside the SEM micrographs suggest that, despite the decreased porosity of samples with the heat treatment, this process is not the best approach to eliminate the H_2_ gas generated from the oxidation of Al.

Kim and Kang [95] focused on optimizing the l/s ratio, NaOH concentration, and particle size of vitrified MIBA (thermal treatment with undisclosed temperature, but expected to be over 1400 °C) in alkali-activated specimens. Naturally, the process of vitrification resulted in a highly amorphous MIBA as demonstrated by the broadened profile in the XRD analysis. As the average particle size decreased from 100–150 µm to <45 µm, by submitting the material to a ball milling process, the 3-day compressive strength increased from ~120 MPa to ~160 MPa, respectively, for an l/s ratio of 0.13. For a fixed particle size of 45 µm, increasing the NaOH concentration from 14 M to 25 M led to an increase in 1-day compressive strength of ~40 MPa to ~160 MPa, respectively, wherein stabilization occurred after 20 M concentration. Similar high strength was achieved more recently in a study attempting to recreate the vitreous fraction of the product of MSW incineration via a high temperature vitrification process [156]. Mixes with solid to liquid ratios of 2.5, using vitrified MIBA and silica fume, resulted in 28-day compressive strengths of 90–120 MPa, which increased with the use of K_2_SiO_3_ for the same K_2_O/SiO_2_ ratio.

#### 4.4.2. Defoaming Process

Huang et al. [137] studied different types of pre-treatments to improve the mechanical performance of alkali-activated MIBA, such as a defoaming process, calcination up to 1050 °C and addition of calcium-containing components (GGBS and slaked lime). The former treatment envisages the complete reaction of Al with the NaOH solution, thereby releasing all of the H_2_ gas. This was carried out by mixing MIBA with the NaOH solution 3 h prior to its use in mortar production. Other treatments involved heating (ranging from 700 °C to 1050 °C). The defoaming technique led to a 28-day compressive strength increase from 2.4 MPa to 8.4 MPa. MIBA that has been heat-treated alongside defoaming exhibited higher compressive strength (~14 MPa after 28 days). FT-IR analysis suggested the presence of C-S-H, C-A-S-H and some carbonates.

#### 4.4.3. Pressing Technique

Qiao et al. [107] evaluated mixes with untreated and thermally treated MIBA at 880 °C activated with Ca(OH)_2_ and with minimum water content in pressed specimens. For a water to solid ratio of 0.20, the authors obtained specimens with 28-day compressive strength of about 7 MPa. When thermally treated MIBA were used, the strength increased to 15 MPa. This strength gain was due to the low moisture of the pressed samples, which decreased the porosity and consequently increased the contact surface areas between the reaction products thereby promoting strength development. Because of the formation of new mineralogical phases as a result of the thermal treatment, conditions became ideal for the reaction between dissolved amorphous SiO_2_ and CaO to produce C-S-H. Even though H_2_ gas formation was still observed, the faster strength development due to pressing may have led to a strong enough microstructure with fast setting and stiffening capable of withstanding the expansion-inducing pressure of H_2_ [157].

Rożek et al. [89] also used a 5 MPa compaction technique in hydrothermally treated samples at 180 °C for 10 h and with modified CaO/SiO_2_ molar ratio of 0.83 by using SiO_2_ or CaO as partial replacement of MIBA. This method led to a significant improvement from 24 MPa to 75 MPa in specimens containing MIBA rich in amorphous phases, which led to a greater dissolution of SiO_2_ and Al_2_O_3_.

### 4.5. Microstructure

Lancellotti et al. [96] concluded that reactive Si/Al ratio is an important parameter to be considered for a proper AAM formulation and that it is essential to determine the quantity of both amorphous Si- and Al-bearing phases in the precursor and the alkaline solutions to understand the potential degree of polymerization. By determining the quantity of potentially reactive aluminosilicate fraction in MIBA, the authors concluded that there is a significant difference between the reactive Si/Al ratio of the MIBA-based AAM due to the variability of crystalline and amorphous fractions with a different degree of reactivity. In addition, the total Si/Al ratio of the sample was between 2.5 and 3.5. According to the study of Davidovits [158], the physical characteristics of hardened geopolymer is significantly affected by the Si/Al ratio, and the mixture can be used in concrete when the ratio is lower than three.

Giro-Paloma et al. [91] performed SEM analysis on alkali-activated MIBA paste and reported a low-density microstructure in spite of the presence of relatively dense inorganic polymers. Additionally, there were unreacted MIBA particles even though the samples were cured for 15 days. The authors also analyzed alkali-activated MIBA paste via FT-IR, the results of which showed a peak at 1000 cm^−1^, related to Si-O-Al and Si-O-Si bonds. Similar findings were made in other studies [96,97]. Also, a peak at 875 cm^−1^ was seen due to the presence of calcium carbonate. Furthermore, Zhu et al. [90] showed that about 20% of the alkali-activated MIBA paste was composed of C-S-H and pirssonite. The authors also showed that the chemical structure of C-S-H in the MIBA paste is similar to that of conventional cement paste. However, the MIBA paste has a higher degree of polymerization of silicate-chains. According to XRD diffractograms, most of the crystalline phases in MIBA particles are quartz, calcite (and other calcium carbonate polymorphs), magnetite, and hydroxyapatite. After alkali activation process, other new peaks were observed because of C-(A)-S-H and Na_2_Ca(CO_3_)_2_·_2_H_2_O.

### 4.6. Toxicity

Besides the mechanical and durability-related performance of alkali-activated MIBA, emphasis was also given to the material’s behavior in terms of binding hazardous compounds. Generally, when compared with untreated MIBA, the solidification/stabilization process by alkali activation significantly decreases heavy metal leaching [103,107].

Biswal et al. [159] studied the leaching behavior of alkali-activated MIBA with metakaolin (mass proportion of 20/80). When compared with the sample which comprised of metakaolin alone, which presented a compressive strength of 43.1 MPa, the one containing 20% MIBA exhibited a significant decline in performance (2.8 MPa). The main reason for this decline was attributed to the presence of a relatively large content of Al. Fourier-transform infrared spectroscopy (FT-IR) analysis showed fairly similar spectra between the control sample and the one containing MIBA, but field emission scanning electron microscopy (FE-SEM) suggested somewhat different microstructure. The former revealed highly clustered crystals with spherical granular shape particles with a structure very similar to that of zeolite crystals, whereas the latter was comprised of both spherical particles and sheet-like structures with greater porosity than the control probably due to the formation CO_2_ and/or H_2_ during the reaction. Regarding the leaching test, the authors compared the behavior of unbound MIBA vs. alkali-activated MIBA + metakaolin. As expected, the activated specimens presented overall lower leachability of heavy metals in both abiotic and biotic media, though a spike in Cr release was observed. Nevertheless, the relatively high presence of arsenic and lead rendered the leachate undrinkable according to the World Health Organization standards.

Chen et al. [92] concluded that both MIBA powder and alkali-activated MIBA paste can be classified as non-hazardous waste materials at landfill based on the UK criteria. However, the concentration of the eluted metal parameters of MIBA powder are higher than the criteria of drinking water standard, while the concentration decreased for all the metal parameters in alkali-activated MIBA paste, except for Cu and Cr. Similar findings were observed by Rożek et al. [89].

Jing et al. [104] decreased the leaching potential and porosity of the alkali-activated paste made with MIBA by using hydrothermal processing method under saturated steam pressure (1 MPa) at temperature of 180 °C for 12 h.

According to the study of Krausova et al. [109], the leaching rate of Cd increased after the heat treatment of MIBA. The opposite occurred for Pb.

Galiano et al. [108] also concluded that heavy metals present in FA and MIBA can be stabilized with the use of alkali activation. Furthermore, Giro-Paloma et al. [91] showed that, apart from As, all the other results for heavy metal leaching are lower than that of the standard threshold specified for landfilling.

Monich et al. [160,161] mixed vitrified MIBA (1500 °C for 60 min) with 10% soda lime silica glass and placed the precursors in a alkaline solution of NaOH (concentration of 1 M or 1.5 M) for 3 h. The material was subsequently fired at 800 °C or 900 °C with a heating rate of 10 °C/min and a holding time of 1 h. This technique, based on alkali activation, gelation, foaming, and sintering, led to the formation of a relatively strong (compressive strength between 5.3 MPa and 8.1 MPa) porous glass-ceramics exhibiting low leaching levels (all within the requirements of EU legislation for the acceptance of inert waste in landfills [162]).

## 5. Conclusions

According to the preliminary evaluation made of the main parameters in the literature, it was shown that alkali-activated MIBA is a very new research topic with relatively little amount of information and with high potential for development. From a general perspective, the applicability of MIBA as solid precursor in the production of AAM greatly depends on the treatment before and after the incineration as well as the efficacy of the combustion itself. This whole process influences the chemical composition of MIBA. Even though these ashes may present adequate, albeit extremely variable, amounts of Si- and Al-bearing compounds, these are likely to be present in crystalline phases rather than amorphous ones, which significantly affects the ashes’ reactivity. This variability, as well as the oxide phase equilibrium and pozzolanicity, which are often absent, may affect the practical viability of the material. Considering these factors from an optimization point of view, there is no consensus in the literature regarding the “best” concentration of the alkaline activator and thus determining the ashes’ composition must be carried out on a case to case basis to calculate it. MIBA also typically presents a significant amount of metallic aluminum, which is not fully removed during the ashes’ treatment process before/after incineration (via eddy current separation). This component is the single most important factor negatively influencing the strength development of all MIBA-based AAM, via expansive reactions (H_2_ gas formation) in the plastic state leading to considerable porosity in the hardened state after setting. Several methods have been proposed to eliminate these issues, by pressing the samples during compaction, increasing the mixing time to potentiate H_2_ reactions and heat treating the material. Nonetheless, despite the aforementioned shortcomings, some have managed to use MIBA as the sole precursor in AAM and achieve considerable mechanical performance, thus demonstrating its potential as an aluminosilicate precursor.

## Figures and Tables

**Table 1:** Chemical composition of Portland cement [119].

Portland Cement	Chemical Composition (%)						
	SiO_2_	Al_2_O_3_	Fe_2_O_3_	CaO	MgO	SO_3_	Na_2_O_eq_
Min-Max	18.6–24.4	2.2–7.3	0.2–5.9	61.3–68.7	0.3–4.5	1.7–4.9	0.09–1.2
Mean	21.3	4.5	3.0	63.9	2.0	2.8	0.5

## References

[1] Detphan S., Chindaprasirt P. (2009). Preparation of fly ash and rice husk ash geopolymer. Int. J. Min. Metal. Mater..

[2] Bernal S.A., Rodríguez E.D., de Gutiérrez R.M., Provis J.L., Delvasto S. (2012). Activation of metakaolin/slag blends using alkaline solutions based on chemically modified silica fume and rice husk ash. Waste Biomass Valorization.

[3] Islam A., Alengaram U.J., Jumaat M.Z., Bashar I.I. (2014). The development of compressive strength of ground granulated blast furnace slag-palm oil fuel ash-fly ash based geopolymer mortar. Mater. Des..

[4] Zarina Y., Al Bakri A.M., Kamarudin H., Nizar I.K., Rafiza A. (2013). Review on the various ash from palm oil waste as geopolymer material. Rev. Adv. Mater. Sci..

[5] Ranjbar N., Mehrali M., Alengaram U.J., Metselaar H.S.C., Jumaat M.Z. (2014). Compressive strength and microstructural analysis of fly ash/palm oil fuel ash based geopolymer mortar under elevated temperatures. Constr. Build. Mater..

[6] Salih M.A., Abang Ali A.A., Farzadnia N. (2014). Characterization of mechanical and microstructural properties of palm oil fuel ash geopolymer cement paste. Constr. Build. Mater..

[7] Matalkah F., Darsanasiri A., Abideen S., Balachadra A., Soroushian P. (2017). Alkali-Activation of Non-Wood Biomass Ash: Effects of Ash Characteristics on Concrete Performance. Civ. Eng. J..

[8] Oyebisi S., Ede A., Olutoge F., Ofuyatan O.M., Oluwafemi J. (2018). Influence of Alkali Concentrations on the Mechanical Properties of Geopolymer Concrete. Int. J. Civ. Eng. Technol. (IJCIET).

[9] Castaldelli V., Akasaki J., Melges J., Tashima M., Soriano L., Borrachero M., Monzó J., Payá J. (2013). Use of slag/sugar cane bagasse ash (SCBA) blends in the production of alkali-activated materials. Materials.

[10] Al-Akhras N.M. (2013). Durability of wheat straw ash concrete to alkali-silica reaction. Proc. Inst. Civ. Eng.-Constr. Mater..

[11] Girón R.P., Gil R.R., Suárez-Ruiz I., Fuente E., Ruiz B. (2015). Adsorbents/catalysts from forest biomass fly ash. Influence of alkaline activating agent. Microporous Mesoporous Mater..

[12] Cheah C.B., Samsudin M.H., Ramli M., Part W.K., Tan L.E. (2017). The use of high calcium wood ash in the preparation of Ground Granulated Blast Furnace Slag and Pulverized Fly Ash geopolymers: A complete microstructural and mechanical characterization. J. Clean. Prod..

[13] Matalkah F., Soroushian P., Ul Abideen S., Peyvandi A. (2016). Use of non-wood biomass combustion ash in development of alkali-activated concrete. Constr. Build. Mater..

[14] Bernal S.A., Rodríguez E.D., Kirchheim A.P., Provis J.L. (2016). Management and valorisation of wastes through use in producing alkali-activated cement materials. J. Chem. Technol. Biotechnol..

[15] Alonso M.M., Gascó C., Morales M.M., Suárez-Navarro J.A., Zamorano M., Puertas F. (2019). Olive biomass ash as an alternative activator in geopolymer formation: A study of strength, radiology and leaching behaviour. Cem. Concr. Compos..

[16] Monneron-Gyurits M., Joussein E., Soubrand M., Fondanèche P., Rossignol S. (2018). Valorization of mussel and oyster shells toward metakaolin-based alkaline activated material. Appl. Clay Sci..

[17] Djobo Y.J.N., Elimbi A., Dika Manga J., Djon Li Ndjock I.B. (2016). Partial replacement of volcanic ash by bauxite and calcined oyster shell in the synthesis of volcanic ash-based geopolymers. Constr. Build. Mater..

[18] Singh N.B., Middendorf B. (2020). Geopolymers as an alternative to Portland cement: An overview. Constr. Build. Mater..

[19] Zhou W., Yan C., Duan P., Liu Y., Zhang Z., Qiu X., Li D. (2016). A comparative study of high-and low-Al_2_O_3_ fly ash based-geopolymers: The role of mix proportion factors and curing temperature. Mater. Des..

[20] Payá J., Agrela F., Rosales J., Morales M.M., Borrachero M.V., de Brito J., Agrela F. (2019). 13-Application of alkali-activated industrial waste. New Trends in Eco-efficient and Recycled Concrete.

[21] Zhang Z., Provis J.L., Zou J., Reid A., Wang H. (2016). Toward an indexing approach to evaluate fly ashes for geopolymer manufacture. Cem. Concr. Res..

[22] Palomo A., Fernández-Jiménez A. Alkaline activation, procedure for transforming fly ash into new materials. Part I: Applications. Proceedings of the World of Coal Ash (WOCA) Conference.

[23] Hajimohammadi A., van Deventer J.S. (2017). Characterisation of one-part geopolymer binders made from fly ash. Waste Biomass Valorization.

[24] Choo H., Lim S., Lee W., Lee C. (2016). Compressive strength of one-part alkali activated fly ash using red mud as alkali supplier. Constr. Build. Mater..

[25] Nematollahi B., Sanjayan J., Shaikh F.U.A. (2014). Comparative deflection hardening behavior of short fiber reinforced geopolymer composites. Constr. Build. Mater..

[26] Kurda R., Silvestre J.D., de Brito J., Ahmed H. (2017). Effect of incorporation of high volume of recycled concrete aggregates and fly ash on the strength and global warming potential of concrete. J. Clean. Prod..

[27] Kurda R., Silvestre J.D., de Brito J., Ahmed H. (2018). Optimizing recycled concrete containing high volume of fly ash in terms of the embodied energy and chloride ion resistance. J. Clean. Prod..

[28] Donatello S., Maltseva O., Fernandez-Jimenez A., Palomo A. (2014). The early age hydration reactions of a hybrid cement containing a very high content of coal bottom ash. J. Am. Ceram. Soc..

[29] Font A., Soriano L., de Moraes Pinheiro S.M., Tashima M.M., Monzó J., Borrachero M.V., Payá J. (2020). Design and properties of 100% waste-based ternary alkali-activated mortars: Blast furnace slag, olive-stone biomass ash and rice husk ash. J. Clean. Prod..

[30] Huseien G.F., Tahir M.M., Mirza J., Ismail M., Shah K.W., Asaad M.A. (2018). Effects of POFA replaced with FA on durability properties of GBFS included alkali activated mortars. Constr. Build. Mater..

[31] Li Z., Liu S. (2007). Influence of slag as additive on compressive strength of fly ash-based geopolymer. J. Mater. Civ. Eng..

[32] Aydın S., Baradan B. (2012). Mechanical and microstructural properties of heat cured alkali-activated slag mortars. Mater. Des..

[33] Mehta A., Siddique R. (2018). Sustainable geopolymer concrete using ground granulated blast furnace slag and rice husk ash: Strength and permeability properties. J. Clean. Prod..

[34] Sun Z., Lin X., Vollpracht A. (2018). Pervious concrete made of alkali activated slag and geopolymers. Constr. Build. Mater..

[35] Assi L., Carter K., Deaver E., Anay R., Ziehl P. (2018). Sustainable concrete: Building a greener future. J. Clean. Prod..

[36] Çevik A., Alzeebaree R., Humur G., Niş A., Gülşan M.E. (2018). Effect of nano-silica on the chemical durability and mechanical performance of fly ash based geopolymer concrete. Ceram. Int..

[37] Duan P., Yan C., Zhou W. (2017). Compressive strength and microstructure of fly ash based geopolymer blended with silica fume under thermal cycle. Cem. Concr. Compos..

[38] Daniel A.J., Sivakamasundari S., Nishanth A. (2017). Study on Partial Replacement of Silica Fume Based Geopolymer Concrete Beam Behavior under Torsion. Procedia Eng..

[39] Okoye F.N., Durgaprasad J., Singh N.B. (2016). Effect of silica fume on the mechanical properties of fly ash based-geopolymer concrete. Ceram. Int..

[40] Assi L.N., Deaver E., Ziehl P. (2018). Using sucrose for improvement of initial and final setting times of silica fume-based activating solution of fly ash geopolymer concrete. Constr. Build. Mater..

[41] Okoye F.N., Prakash S., Singh N.B. (2017). Durability of fly ash based geopolymer concrete in the presence of silica fume. J. Clean. Prod..

[42] Kovtun M., Kearsley E.P., Shekhovtsova J. (2015). Dry powder alkali-activated slag cements. Adv. Cem. Res..

[43] Granizo M., Blanco-Varela M., Palomo A. (2000). Influence of the starting kaolin on alkali-activated materials based on metakaolin. Study of the reaction parameters by isothermal conduction calorimetry. J. Mater. Sci..

[44] Longhi M.A., Rodríguez E.D., Bernal S.A., Provis J.L., Kirchheim A.P. (2016). Valorisation of a kaolin mining waste for the production of geopolymers. J. Clean. Prod..

[45] Duxson P., Mallicoat S.W., Lukey G.C., Kriven W.M., van Deventer J.S. (2007). The effect of alkali and Si/Al ratio on the development of mechanical properties of metakaolin-based geopolymers. Colloids Surf. Physicochem. Eng. Aspects.

[46] Shoaei P., Musaeei H.R., Mirlohi F., Narimani zamanabadi S., Ameri F., Bahrami N. (2019). Waste ceramic powder-based geopolymer mortars: Effect of curing temperature and alkaline solution-to-binder ratio. Constr. Build. Mater..

[47] Reig L., Tashima M., Soriano L., Borrachero M., Monzó J., Payá J. (2013). Alkaline activation of ceramic waste materials. Waste Biomass Valorization.

[48] Dimas D.D., Giannopoulou I.P., Panias D. (2009). Utilization of alumina red mud for synthesis of inorganic polymeric materials. Miner. Process. Extr. Metall. Rev..

[49] Gong C., Yang N. (2000). Effect of phosphate on the hydration of alkali-activated red mud–slag cementitious material. Cem. Concr. Res..

[50] Kumar A., Kumar S. (2013). Development of paving blocks from synergistic use of red mud and fly ash using geopolymerization. Constr. Build. Mater..

[51] Ramezanianpour A.A., Kazemian A., Sarvari M., Ahmadi B. (2013). Use of natural zeolite to produce self-consolidating concrete with low portland cement content and high durability. J. Mater. Civ. Eng..

[52] Raggiotti B.B., Positieri M.J., Oshiro Á. (2018). Natural zeolite, a pozzolan for structural concrete. Procedia Struct. Integr..

[53] Li J., Zhang W., Li C., Monteiro P.J.M. (2019). Green concrete containing diatomaceous earth and limestone: Workability, mechanical properties, and life-cycle assessment. J. Clean. Prod..

[54] Yılmaz B., Ediz N. (2008). The use of raw and calcined diatomite in cement production. Cem. Concr. Compos..

[55] Vejmelková E., Koňáková D., Doleželová M., Scheinherrová L., Svora P., Keppert M., Reiterman P., Černý R. (2018). Effect of calcined Czech claystone on the properties of high performance concrete: Microstructure, strength and durability. Constr. Build. Mater..

[56] Tagnit-Hamou A., Petrov N., Luke K. (2003). Properties of concrete containing diatomaceous earth. ACI Mater. J..

[57] Abrão P., Cardoso F., John V. (2019). Evaluation of Portland pozzolan blended cements containing diatomaceous earth. Cerâmica.

[58] Kani E.N., Allahverdi A., Provis J.L. (2012). Efflorescence control in geopolymer binders based on natural pozzolan. Cem. Concr. Compos..

[59] Kani E.N., Allahverdi A. (2009). Effect of chemical composition on basic engineering properties of inorganic polymeric binder based on natural pozzolan. Ceramics-Silikaty.

[60] Lemougna P.N., Wang K., Tang Q., Nzeukou A.N., Billong N., Melo U.C., Cui X. (2018). Review on the use of volcanic ashes for engineering applications. Resour. Conserv. Recy..

[61] Siddique R. (2012). Properties of concrete made with volcanic ash. Resour. Conserv. Recy..

[62] Hossain K.M.A., Lachemi M. (2007). Strength, durability and micro-structural aspects of high performance volcanic ash concrete. Cem. Concr. Res..

[63] Safari Z., Kurda R., Al-Hadad B., Mahmood F., Tapan M. (2020). Mechanical characteristics of pumice-based geopolymer paste. Resour. Conserv. Recy..

[64] Yadollahi M.M., Benli A., Demirboğa R. (2015). The effects of silica modulus and aging on compressive strength of pumice-based geopolymer composites. Constr. Build. Mater..

[65] Almalkawi A.T., Hamadna S., Soroushian P. (2017). One-part alkali activated cement based volcanic pumice. Constr. Build. Mater..

[66] Kourti I., Devaraj A.R., Bustos A.G., Deegan D., Boccaccini A.R., Cheeseman C.R. (2011). Geopolymers prepared from DC plasma treated air pollution control (APC) residues glass: Properties and characterisation of the binder phase. J. Hazard. Mater..

[67] Martinez-Lopez R., Ivan Escalante-Garcia J. (2016). Alkali activated composite binders of waste silica soda lime glass and blast furnace slag: Strength as a function of the composition. Constr. Build. Mater..

[68] Liu Y., Shi C., Zhang Z., Li N. (2019). An overview on the reuse of waste glasses in alkali-activated materials. Resour. Conserv. Recy..

[69] Tashima M., Soriano L., Borrachero M., Monzó J., Cheeseman C., Payá J. (2012). Alkali activation of vitreous calcium aluminosilicate derived from glass fiber waste. J. Sustain. Cem.-Based Mater..

[70] Pascual A.B., Tognonvi M.T., Tagnit-Hamou A. (2014). Waste glass powder-based alkali-activated mortar. Int. J. Res. Eng. Technol..

[71] Puertas F., Torres-Carrasco M. (2014). Use of glass waste as an activator in the preparation of alkali-activated slag. Mechanical strength and paste characterisation. Cem. Concr. Res..

[72] Torres-Carrasco M., Puertas F. (2015). Waste glass in the geopolymer preparation. Mechanical and microstructural characterisation. J. Clean. Prod..

[73] Cherian C., Siddiqua S. (2019). Pulp and Paper Mill Fly Ash: A Review. Sustainability.

[74] Yang K.H., Lo C.W., Huang J.S. (2013). Production and properties of foamed reservoir sludge inorganic polymers. Cem. Concr. Compos..

[75] Guo X., Shi H., Dick W. (2010). Use of heat-treated water treatment residuals in fly ash-based geopolymers. J. Am. Ceram. Soc..

[76] Banfill P., Frias M. (2007). Rheology and conduction calorimetry of cement modified with calcined paper sludge. Cem. Concr. Res..

[77] Santa R.A.A.B., Bernardin A.M., Riella H.G., Kuhnen N.C. (2013). Geopolymer synthetized from bottom coal ash and calcined paper sludge. J. Clean. Prod..

[78] Li R., Zhang B., Wang Y., Zhao Y., Li F. (2019). Leaching potential of stabilized fly ash from the incineration of municipal solid waste with a new polymer. J. Environ. Manag..

[79] Ryu G.S., Lee Y.B., Koh K.T., Chung Y.S. (2013). The mechanical properties of fly ash-based geopolymer concrete with alkaline activators. Constr. Build. Mater..

[80] Shiota K., Nakamura T., Takaoka M., Aminuddin S.F., Oshita K., Fujimori T. (2017). Stabilization of lead in an alkali-activated municipal solid waste incineration fly ash–Pyrophyllite-based system. J. Environ. Manag..

[81] Sofi M., van Deventer J.S.J., Mendis P.A., Lukey G.C. (2007). Engineering properties of inorganic polymer concretes (IPCs). Cem. Concr. Res..

[82] Yakubu Y., Zhou J., Ping D., Shu Z., Chen Y. (2018). Effects of pH dynamics on solidification/stabilization of municipal solid waste incineration fly ash. J. Environ. Manag..

[83] Lach M., Mierzwinski D., Korniejenko K., Mikula J., Hebda M. (2018). Geopolymers as a material suitable for immobilization of fly ash from municipal waste incineration plants. J. Air Waste Manag. Assoc..

[84] Shao Y., Hou H., Wang G., Wan S., Zhou M. (2014). Characteristics of the stabilized/solidified municipal solid wastes incineration fly ash and the leaching behavior of Cr and Pb. Front. Environ. Sci. Eng..

[85] Jin M., Zheng Z., Sun Y., Chen L., Jin Z. (2016). Resistance of metakaolin-MSWI fly ash based geopolymer to acid and alkaline environments. J. Non-Cryst. Solids.

[86] Ferone C., Colangelo F., Messina F., Santoro L., Cioffi R. (2013). Recycling of Pre-Washed Municipal Solid Waste Incinerator Fly Ash in the Manufacturing of Low Temperature Setting Geopolymer Materials. Materials.

[87] Aliabdo A.A., Abd Elmoaty A.E.M., Emam M.A. (2019). Factors affecting the mechanical properties of alkali activated ground granulated blast furnace slag concrete. Constr. Build. Mater..

[88] Zhu W.P., Chen X., Zhao A.Q., Struble L.J., Yang E.H. (2019). Synthesis of high strength binders from alkali activation of glass materials from municipal solid waste incineration bottom ash. J. Clean. Prod..

[89] Rożek P., Król M., Mozgawa W. (2019). Solidification/stabilization of municipal solid waste incineration bottom ash via autoclave treatment: Structural and mechanical properties. Constr. Build. Mater..

[90] Zhu W., Chen X., Struble L., Yang E. (2018). Characterization of calcium-containing phases in alkali-activated municipal solid waste incineration bottom ash binder through chemical extraction and deconvoluted Fourier transform infrared spectra. J. Clean. Prod..

[91] Giro-Paloma J., Maldonado-Alameda A., Formosa J., Barbieri L., Chimenos J.M., Lancellotti I. (2017). Geopolymers based on the valorization of municipal solid waste incineration residues. IOP Conf. Ser. Mater. Sci. Eng..

[92] Chen Z., Liu Y., Zhu W., Yang E.-H. (2016). Incinerator bottom ash (IBA) aerated geopolymer. Constr. Build. Mater..

[93] Zhu W., Chen X., Struble L., Yang E. Feasibility study of municipal solid waste incinerator bottom ash as geopolymer precursor. Proceedings of the Fourth International Conference on Sustainable Construction Materials and Technologies.

[94] Song Y., Li B., Yang E., Liu Y., Ding T. (2015). Feasibility study on utilization of municipal solid waste incineration bottom ash as aerating agent for the production of autoclaved aerated concrete. Cem. Concr. Compos..

[95] Kim Y., Kang S. (2014). Characterization of geopolymer made of municipal solid waste incineration ash slag. J. Korean Cryst. Growth Cryst. Technol..

[96] Lancellotti I., Ponzoni C., Barbieri L., Leonelli C. (2013). Alkali activation processes for incinerator residues management. Waste Manag..

[97] Onori R., Will J., Hoppe A., Polettini A., Pomi R., Boccaccini A., Kriven W.M., Gyekenyesi A.L., Wang J., Widjaja S., Singh D. (2011). Bottom ash-based geopolymer materials: Mechanical and environmental properties. Developments in Strategic Materials and Computational Design II: Ceramic Engineering and Science Proceedings.

[98] Qiao X., Tyrer M., Poon C., Cheeseman C. (2008). Characterization of alkali-activated thermally treated incinerator bottom ash. Waste Manag..

[99] Huang G., Ji Y., Li J., Zhang L., Liu X., Liu B. (2019). Effect of activated silica on polymerization mechanism and strength development of MSWI bottom ash alkali-activated mortars. Constr. Build. Mater..

[100] Huang G., Ji Y., Zhang L., Li J., Hou Z. (2018). The influence of curing methods on the strength of MSWI bottom ash-based alkali-activated mortars: The role of leaching of OH- and free alkali. Constr. Build. Mater..

[101] Liu Y., Sidhu K., Chen Z., Yang E. (2018). Alkali-treated incineration bottom ash as supplementary cementitious materials. Constr. Build. Mater..

[102] Wongsa A., Boonserm K., Waisurasingha C., Sata V., Chindaprasirt P. (2017). Use of municipal solid waste incinerator (MSWI) bottom ash in high calcium fly ash geopolymer matrix. J. Clean. Prod..

[103] Garcia-Lodeiro I., Carcelen-Taboada V., Fernández-Jiménez A., Palomo A. (2016). Manufacture of hybrid cements with fly ash and bottom ash from a municipal solid waste incinerator. Constr. Build. Mater..

[104] Jing Z., Jin F., Yamasaki N., Ishida E. (2007). Hydrothermal synthesis of a novel tobermorite-based porous material from municipal incineration bottom ash. Ind. Eng. Chem. Res..

[105] Penilla R., Bustos A., Elizalde S. (2003). Zeolite synthesized by alkaline hydrothermal treatment of bottom ash from combustion of municipal solid wastes. J. Am. Ceram. Soc..

[106] Xuan D., Tang P., Poon C. (2019). MSWIBA-based cellular alkali-activated concrete incorporating waste glass powder. Cem. Concr. Compos..

[107] Qiao X.C., Tyrer M., Poon C.S., Cheeseman C.R. (2008). Novel cementitious materials produced from incinerator bottom ash. Resour. Conserv. Recy..

[108] Galiano Y.L., Pereira C.F., Vale J. (2011). Stabilization/solidification of a municipal solid waste incineration residue using fly ash-based geopolymers. J. Hazard. Mater..

[109] Krausova K., Cheng T.W., Gautron L., Dai Y.S., Borenstajn S. (2012). Heat treatment on fly and bottom ash based geopolymers: Effect on the immobilization of lead and cadmium. Int. J. Environ. Sci. Dev..

[110] Silva R.V., de Brito J., Lynn C.J., Dhir R.K. (2017). Use of municipal solid waste incineration bottom ashes in alkali-activated materials, ceramics and granular applications: A review. Waste Manag..

[111] Haukohl J., Kristiansen T. (1996). Waste Incineration.

[112] Dhir R.K., De Brito J., Lynn C.J., Silva R.V. (2018). Sustainable Construction Materials: Municipal Incinerator Bottom Ash.

[113] Shen H., Forssberg E. (2003). An overview of recovery of metals from slags. Waste Manag..

[114] Joseph A.M., Snellings R., Van den Heede P., Matthys S., De Belie N. (2018). The use of municipal solid waste incineration ash in various building materials: A Belgian point of view. Materials.

[115] Seleem H.E.D.H., Rashad A.M., Elsokary T. (2011). Effect of elevated temperature on physico-mechanical properties of blended cement concrete. Constr. Build. Mater..

[116] Dinakar P., Babu K.G., Santhanam M. (2008). Durability properties of high volume fly ash self compacting concretes. Cem. Concr. Compos..

[117] Thomas M.D.A., Bamforth P.B. (1999). Modelling chloride diffusion in concrete: Effect of fly ash and slag. Cem. Concr. Res..

[118] Richartz W. (1986). Effect of the K_2_O content and degree of sulfatization on the setting and hardening of cement. Zement-Kalk-Gips.

[119] Kosmatka S., Kerkhoff B.C., Panarese W. (2002). Design and Control of Concrete Mixtures.

[120] Saccani A., Sandrolini F., Andreola F., Barbieri L., Corradi A., Lancellotti I. (2005). Influence of the pozzolanic fraction obtained from vitrified bottom-ashes from MSWI on the properties of cementitious composites. Mater. Struct..

[121] Tang P., Florea M., Spiesz P., Brouwers H. (2016). Application of thermally activated municipal solid waste incineration (MSWI) bottom ash fines as binder substitute. Cem. Concr. Compos..

[122] Bertolini L., Carsana M., Cassago D., Curzio A.Q., Collepardi M. (2004). MSWI ashes as mineral additions in concrete. Cem. Concr. Res..

[123] EN 197–1, Cement (2011). Composition, Specifications and Conformity Criteria for Common Cements.

[124] (2007). RSC TiO2: Uses of titanium dioxide. Titanium Dioxide Photocatalysis: Uses of Titanium Dioxide.

[125] Odler I., Schmidt O. (2006). Structure and properties of Portland cement clinker doped with zinc oxide. J. Am. Ceram. Soc..

[126] Engelsen C. (2007). Effect of Mineralizers in Cement Production- State of the Art.

[127] Jackson J., Hewlett P.C. (1998). Portland cement: Classification and manufacture. Lea’s Chemistry of Cement and Concrete.

[128] Sprung S. (1985). Technological Problems in Pyroprocessing Cement Clinker: Cause and Solution.

[129] Bhatty I. (1995). Role of minor elements in cement: Manufacture and use. International Report of the Portland Cement Association.

[130] Provis J.L. (2018). Alkali-activated materials. Cem. Concr. Res..

[131] Provis J.L., Provis J.L., van Deventer J.S.J. (2009). 4-Activating solution chemistry for geopolymers. Geopolymers.

[132] Golek L. (2019). Glass powder and high-calcium fly ash based binders - Long term examinations. J. Clean. Prod..

[133] Samarakoon M.H., Ranjith P.G., De Silva V.R.S. (2020). Effect of soda-lime glass powder on alkali-activated binders: Rheology, strength and microstructure characterization. Constr. Build. Mater..

[134] Zhu W.P., Chen X., Struble L.J., Yang E.H. (2019). Quantitative characterization of aluminosilicate gels in alkali-activated incineration bottom ash through sequential chemical extractions and deconvoluted nuclear magnetic resonance spectra. Cem. Concr. Compos..

[135] Huang G., Ji Y., Zhang L., Li J., Hou Z. (2018). Advances in understanding and analyzing the anti-diffusion behavior in complete carbonation zone of MSWI bottom ash-based alkali-activated concrete. Constr. Build. Mater..

[136] Cristelo N., Segadaes L., Coelho J., Chaves B., Sousa N.R., Lopes M.D. (2020). Recycling municipal solid waste incineration slag and fly ash as precursors in low-range alkaline cements. Waste Manag..

[137] Huang G., Yang K., Chen L., Lu Z., Sun Y., Zhang X., Feng Y., Ji Y., Xu Z. (2020). Use of pretreatment to prevent expansion and foaming in high-performance MSWI bottom ash alkali-activated mortars. Constr. Build. Mater..

[138] Provis J.L., van Deventer J.S.J. (2014). Alkali Activated Materials-State-of-the-Art Report.

[139] Rowles M., O’Connor B. (2003). Chemical optimisation of the compressive strength of aluminosilicate geopolymers synthesised by sodium silicate activation of metakaolinite. J. Mater. Chem..

[140] Lancellotti I., Kamseu E., Michelazzi M., Barbieri L., Corradi A., Leonelli C. (2010). Chemical stability of geopolymers containing municipal solid waste incinerator fly ash. Waste Manag..

[141] Part W.K., Ramli M., Cheah C.B. (2015). An overview on the influence of various factors on the properties of geopolymer concrete derived from industrial by-products. Constr. Build. Mater..

[142] Maldonado-Alameda A., Giro-Paloma J., Svobodova-Sedlackova A., Formosa J., Chimenos J.M. (2020). Municipal solid waste incineration bottom ash as alkali-activated cement precursor depending on particle size. J. Clean. Prod..

[143] Görhan G., Kürklü G. (2014). The influence of the NaOH solution on the properties of the fly ash-based geopolymer mortar cured at different temperatures. Compos. Part B Eng..

[144] Sukmak P., Horpibulsuk S., Shen S.-L. (2013). Strength development in clay–fly ash geopolymer. Constr. Build. Mater..

[145] Somna K., Jaturapitakkul C., Kajitvichyanukul P., Chindaprasirt P. (2011). NaOH-activated ground fly ash geopolymer cured at ambient temperature. Fuel.

[146] Ridtirud C., Chindaprasirt P., Pimraksa K. (2011). Factors affecting the shrinkage of fly ash geopolymers. Int. J. Miner. Metall. Mater..

[147] Guo X., Shi H., Dick W.A. (2010). Compressive strength and microstructural characteristics of class C fly ash geopolymer. Cem. Concr. Compos..

[148] Law D.W., Adam A.A., Molyneaux T.K., Patnaikuni I., Wardhono A. (2014). Long term durability properties of class F fly ash geopolymer concrete. Mater. Struct..

[149] He J., Jie Y., Zhang J., Yu Y., Zhang G. (2013). Synthesis and characterization of red mud and rice husk ash-based geopolymer composites. Cem. Concr. Compos..

[150] Nazari A., Bagheri A., Riahi S. (2011). Properties of geopolymer with seeded fly ash and rice husk bark ash. Mater. Sci. Eng. A.

[151] Songpiriyakij S., Kubprasit T., Jaturapitakkul C., Chindaprasirt P. (2010). Compressive strength and degree of reaction of biomass- and fly ash-based geopolymer. Constr. Build. Mater..

[152] Yusuf M., Johari M., Ahmad Z., Maslehuddin M. (2014). Impacts of silica modulus on the early strength of alkaline activated ground slag/ultrafine palm oil fuel ash based concrete. Mater. Struct..

[153] Ahmari S., Zhang L. (2012). Production of eco-friendly bricks from copper mine tailings through geopolymerization. Constr. Build. Mater..

[154] Coelho J., Chaves B., Lopes M.L., Segadães L., Cristelo N. Stabilizing municipal solid waste incineration residues with alternative binders, 5th. Proceedings of the International Conference Wastes.

[155] Huang G., Yang K., Sun Y., Lu Z., Zhang X., Zuo L., Feng Y., Qian R., Qi Y., Ji Y. (2020). Influence of NaOH content on the alkali conversion mechanism in MSWI bottom ash alkali-activated mortars. Constr. Build. Mater..

[156] Ascensao G., Marchi M., Segata M., Faleschini F., Pontikes Y. (2020). Reaction kinetics and structural analysis of alkali activated Fe-Si-Ca rich materials. J. Clean. Prod..

[157] Zhu W.P., Teoh P.J., Liu Y.Q., Chen Z.T., Yang E.H. (2019). Strategic utilization of municipal solid waste incineration bottom ash for the synthesis of lightweight aerated alkali-activated materials. J. Clean. Prod..

[158] Davidovits J. (1991). Geopolymers-Inorganic polymeric new materials. J. Therm. Anal..

[159] Biswal B.K., Zhu W., Yang E.H. (2020). Investigation on Pseudomonas aeruginosa PAO1-driven bioleaching behavior of heavy metals in a novel geopolymer synthesized from municipal solid waste incineration bottom ash. Constr. Build. Mater..

[160] Monich P.R., Desideri D., Bernardo E. (2019). Low temperature upcycling of vitreous byproduct of the MSW plasma processing into multifunctional porous glass-ceramics. Adv. Appl. Ceram..

[161] Monich P.R., Dogrul F., Lucas H., Friedrich B., Bernardo E. (2019). Strong porous glass-ceramics from alkali activation and sinter-crystallization of vitrified MSWI bottom ash. Detritus.

[162] (2003). CEU Council Decision of 19 December 2002 establishing criteria and procedures for the acceptance of waste at landfills pursuant to Article 16 of and Annex II to Directive 1999/31/EC. Off. J. Eur. Communities.

